# Isotope Mass Spectrometry in the Solar System Exploration

**DOI:** 10.5702/massspectrometry.S0076

**Published:** 2018-09-28

**Authors:** Shoichiro Yokota

**Affiliations:** 1 Department of Earth and Space Science, Graduate School of Science, Osaka University, 1–1 Machikaneyama-cho, Toyonaka 560–0043, Japan

**Keywords:** mass spectrometers, spaceborne instruments, planetary exploration missions

## Abstract

Isotope analyses using mass spectrometers have been frequently utilized in the laboratories for the earth planetary science and other scientific and industrial fields. In order to conduct *in-situ* measurements of compositions and isotope ratios around planets and moons, mass spectrometers onboard spacecraft have also been developed. Ion and electron instruments on orbiters have provided much outputs for the space and planetary science since the early days and mass spectrometers on landers and rovers have recently performed isotope analyses on planetary bodies. We review spaceborne mass spectrometers, instrumentations, and observation results. Starting with spaceborne ion instruments to measure three distribution functions as well as mass for the space plasma physics, mass spectrometers have evolved to recent high-mass-resolution instruments for solar system exploration missions.

## INTRODUCTION

Spaceborne ion (and electron) instruments had been initially developed for the space plasma physics. Design details of the instruments were optimized for measuring space plasma populations that belong to the solar wind and terrestrial and planetary plasma environments. Since the space plasma is collisionless and thus does not necessarily form Maxwellian distributions as the air, scientific objectives of space plasma explorers fundamentally require rapid measurements of three-dimensional distribution functions with adequate phase-space resolutions. Ion observation in space demonstrated that ions of the solar wind and Earth’s magnetosphere contained large admixture of heavy ions that are critical to many interaction processes. In addition, the ion mass information is essential for establishing their sources of origin. Therefore, ion composition measurements have been also an object of great interest and have been improved tremendously in recent years.

**Figure 1:**
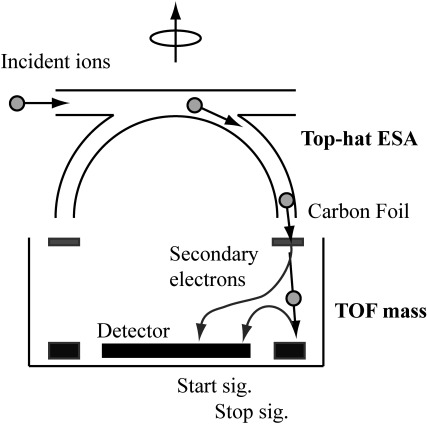
Fig. 1. Schematic view of a typical ion energy mass spectrometer for the space plasma observation. The cylindrically symmetric instruments consists of a top-hat electrostatic analyzer (ESA) for energy/charge measurements and a time-of-flight (TOF) mass/charge analyzer. Secondary electrons are used for start and stop signals.

For three-dimensional energy analyses of low-energy ions (and electrons), the top-hat electrostatic method using spherical deflectors^1)^ or toroidal deflectors^2)^ have usually been applied because of its large geometric factor and uniform angular response while requiring relatively low resources (see Fig. 1). Compete spherical field-of-view (FOV) of 4π steradian is achieved by the 360° aperture of the sensors and the spin motion of spacecraft. The technique of electrically scanning FOVs has recently employed for the fast time resolution^3)^ or application onboard three-axis stabilized spacecraft.^4)^

On the other hand, composition measurements in space, especially near the Earth, Mars, Venus, other planets, moons, and asteroids are of great interest. Several types of mass spectrometers have been utilized in combination with top-hat energy spectrometers.^1,2)^ First, magnetic mass analyses had been actually developed for space plasma measurements.^5)^ The ion mass measurements in space have been followed by another type of mass spectrometers using electric and magnetic fields,^6–8)^ and several sorts of time-of-flight (TOF) mass spectrometers.^9)^ In recent years, the techniques of the planetary landers and rovers have considerably advanced, which are equipped with high-resolution mass spectrometers using quadrupole,^10)^ double focusing, and reflectron^11)^ techniques. The measurement principles of the instruments are similar to that used in laboratories.

Table 1 summarizes spaceborne mass spectrometers though it does not cover all the instruments. We will show different ion mass spectrometers on spacecraft and the observation results and that on lander and revers after briefly describing limitations and requirements of spaceborne instruments. Future Japanese exploration missions with mass spectrometers will be also presented.

**Table 1:** Table 1. Spaceborne mass spectrometers on orbiters, landers, and rovers.

Type	Spacecraft	Energy range & mass resolution	References
Wien filter with energy analyses (EA)	Explorer 34, 1971-089 A, CRRES	< ∼10 s keV/q M/ΔM<∼5	5, 14, 15
Double focusing with EA	GEOS, ISEE 1, DE 1, AMPTE/CCE, AKEBONO, GEOTAIL, POLAR	< ∼10 s keV/q	16–20, 24, 25
		M/ΔM<∼10	
Straight time-of-flight (TOF) with EA	AMPTE/CCE, GIOTTO, CRESS, CLUSTER, ARASE	< ∼30–200 keV/q	12, 13, 28–31
		M/ΔM <10	
Linear-electric-field TOF with EA	KAGUYA, CASSINI, BEPI-COLOMBO	< ∼30 keV/q	4, 34, 35
		M/ΔM∼20–100	
Double focusing	GIOTTO, ROSETTA	M/ΔM∼100–3000	11, 37
Reflectron	GIOTTO, VEGA, ROSETTA	M/ΔM∼100–500	11, 37, 38
Quadrupole	PIONEER VENUS, GALILEO, NOZOMI, CASSINI, LADEE, MAVEN, CURIOSITY	M/ΔM∼10 s–100	10, 41–46

## SPACE-BORNE SCIENTIFIC INSTRUMENTS

In the space exploration missions, their scientific objectives strictly define the performance requirements of the scientific instruments, such as resolutions, sensitivities, and so on. Each instrument is developed to satisfy the requirements while the payload resources are severely limited. Small, light, and micropower instruments are suitable for the payload on spacecraft. Note that the resources of recent typical ion energy mass spectrometers which are often cylindrically symmetric as shown in Fig. 1 are 5–10 kg, ~ϕ20×∼50 cm, and 10–20 W power consumption.^12,13)^ Today’s spacecraft cannot bring laboratory mass spectrometers which almost occupy the room. Scientific instruments of substantial resources are sometimes acceptable in the case that its observation objectives are much important in its mission, while instruments of smaller resources are more accepted.

One of the requirements for spaceborne scientific instruments is robustness for the launch. Spacecraft and its payload are subject to intense acoustic environments during launch, inducing high levels of vibration in structural elements and equipment. Moreover, flight control systems and elastic structural interactions with propulsion systems might provide low-frequency, high-deflection flight instabilities. For all instruments, therefore, it is needed to evaluate all aspects of structural dynamics including vibration vibroacoustic, modal characteristics and shock testing. Note that robust structures for vibration are against reducing the resources.

Robustness for space environment is also indispensable, especially for the thermal design. Spacecraft in space is heated by the solar radiation, and planetary albedo and infrared radiation, while it is cooled *via* radiation to space of absolute zero temperature. Thus, thermal models are investigated numerically and experimentally to clarify whether it keeps the temperature within the acceptable range especially regarding the inside electronics. The thermal model of each scientific instrument is also evaluated independently and in combination of that of the spacecraft. If the performance strongly depends on the temperature, specific thermal structures and functions including heaters and coolers are applied.

Another point is the radiation dose caused by incident flux of mainly high-energy protons and electrons in space. Electronic devices, in particular semiconductors, might malfunction and/or fail due to the radiation in space. Therefore, all electronic devices are selected according to some criteria of the radiation tolerance, depending on the planned trajectories.

Preparing high-voltage power supplies (HVPSs) for a space application is briefly described here, which are indispensable for all types of mass spectrometers. The HVPSs work at ∼10^2^∼10^4^ V in vacuum differently from that of laboratories used in the atmosphere. The technique for preventing electric discharge, thermal situations, and outgassing are different between in vacuum and in the air. Therefore, the design, fabrication, and handling of the HVPSs are carefully considered for developing space-borne mass spectrometers. Baking and insulating material coating are necessary in some cases. It is needed to confirm in thermal vacuum tests with several cycles that the HVPSs of the flight model function well without electrical discharges.

## MAGNETIC SECTOR MASS SPECTROMETERS

In the late 1960s, mass analyzers using crossed rectilinear electric and magnetic fields, so-called ‘Wien filter,’ were developed to measure the solar wind composition up to 5 keV/q for the Explorer 34 mission,^5)^ energetic heavy ions of below 12 keV/q in the ring current for the polar-orbiting satellite 1971-089 A,^14)^ and ions up to 35 keV/q in the radiation belts for the Combined Release and Radiation Effects Satellite (CRRES) mission.^15)^ Another type of electric and magnetic-field mass spectroscopy, ‘double focusing,’ was also developed, which produced wide energy range and thus was extensively used for measurements of hot space plasma of 10 s keV/q.^6–8)^ In the double focusing technique, the ion rays which diverge slightly in angle and energy at the sensor entrance are focused at the detector. Such mass spectrometers played a significant role in measurements of hot magnetospheric ions in the Geodetic Earth Orbiting Satellite (GEOS) missions first,^16)^ and then were applied in the International Sun-Earth Explorer (ISEE) 1,^17)^ Dynamics Explorer (DE) 1,^18)^ and Active Magnetospheric Particle Tracer Explorers/Charge Composition Explorer (AMPTE/CCE) spacecraft.^19)^

In the 1980s, position-sensitive detectors were developed, which were combined with the double focusing technique for simultaneous measurements of mass distributions, resulting in high time resolution. After a similar mass analyzer was used in the AKEBONO mission,^20)^ more advanced type of the mass spectrometers were developed, such as dual mass channels,^21)^ simultaneous spectrographic image of mass/charge values using the Mattauch–Herzog principle,^22)^ and 360° simultaneous image with cylindrically symmetric geometry.^23)^ As the latest version, the double-focusing mass spectrometers combined with top-hat energy analyzers were developed for the space plasma observation by the GEOTAIL^24)^ and POLAR spacecraft.^25)^

Mass resolution of the above magnetic sector instruments for the space plasma observation is enough high to separate ion species of the solar wind and Earth’s magnetosphere, such as H^+^, He^++^, He^+^, and O^+^. The inside magnetic field, however, is uniform over the section of mass analyses and is terminated by high permeability material and heavy iron yokes. Although cylindrically symmetric magnetic analyzers creating a self-contained loop of the magnetic field need no yokes, the whole of the magnetic system poses substantial resources. Moreover, application of a permanent magnet causes the worse mass resolution with increasing ion energy and hence limits the energy range. Although an ion mass spectrometer on the GEOTAIL spacecraft is equipped with electromagnets,^26)^ such type instruments have rarely been employed due to its large resources. Since the right trade-off between particle throughput and resolution is critical in electric and magnetic fields mass spectrometry, the downsizing of the sensors is difficult especially when minor ion species are of interest. Critical properties of the ion optics such as mass dispersion and focusing cause more complicated structures and more resources for a given sensitivity, compared to ion energy analyzers and TOF instruments. In recent missions for the space plasma observation, TOF mass spectrometers have been preferably utilized because of simultaneous detection all over the mass range, large throughout, low resources, and simple structures.

## TOF MASS SPECTROMETERS

**Figure 2:**
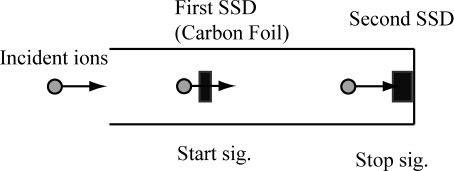
Fig. 2. Schematic view of a time-of-flight (TOF) mass spectrometer for high-energy particles. Solid state detectors (SSDs) measure start and stop signals and total energies.

TOF measurement techniques were originally utilized for a variety of laboratory applications and then were extended to spaceborne instruments as reviewed in detail.^9)^ Typical ion spectrometers employ top-hat electrostatic energy/charge analyses, TOF mass/charge (corresponding to velocity) analyses, and total energy analyses by solid state detectors (SSDs), providing all the three ion parameters, energy, charge, and mass.^27)^ Start signals are derived from energy proportional signals when incident ions pass though the first SSD, while stop signals are generated when the ions hit the second SSD that also conducts total energy analyses (see Fig. 2). Measured flight times and the known straight path lengths between the two SSDs provide the velocities of incident ions. Thin foils were used instead of the first SSD to increase the mass resolution for the AMPTE/CCE mission,^28)^ because such foils cause relatively small angular and energy dispersions. In this case, the start signals are provided by secondary electrons emitted from thin foils due to the ion passage. The SSD technique was not applied to the low-energy ion measurements because the thin window or dead layer on the detector surface creates an ion detection threshold of a few kilo electronvolts per nucleon. Application of ultra-thin (∼1 μg/cm^2^) carbon foils and microchannel plates (MCPs), however, overcame such lower energy limits and opened a way of measuring low-energy ions. Note that a post-acceleration voltage of 5–10 kV applied to the foils is needed for increasing the transmission efficiency and mass resolution.

In addition to the adequate mass resolution and sensitivity with low resources, the TOF method enables noise rejection, for example against penetrating background radiation, by using a coincidence between start and stop pulses within the long flight time. Thus, the TOF mass spectrometers using ultra-thin carbon foils are the most frequently applied for the space plasma observation, first for the GIOTTO mission that went to comet Halley,^29)^ and later for the CRESS mission.^30)^ A cylindrically symmetric ion mass spectrometer combined with a 360° FOV top-hat energy analyzer was designed,^2)^ and advanced to that for CLUSTER mission.^31)^ Recent Japanese spacecraft, ARASE, also holds such ion instruments and successfully obtained mass spectra in the radiation belts.^8,9)^ The flight time critically depends on the energy degradation and angular scattering of incident particles due to the passage through ultra-thin carbon foil. Thus, the mass resolution is limited up to M/ΔM∼10 while sufficient to discriminate main ion species in the solar wind and Earth’s magnetosphere.

**Figure 3:**
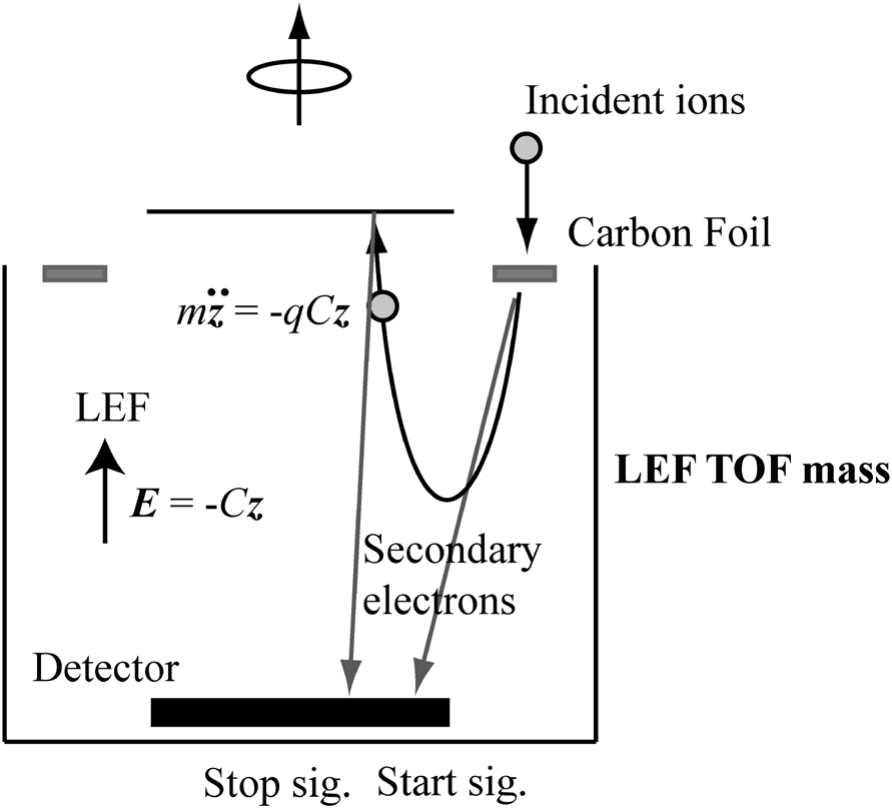
Fig. 3. Schematic view of a linear-electric-field (LEF) TOF mass spectrometer. In the LEF generated in the sensor, the motion of incident ions are expressed by a simple harmonic oscillator.

In order to improve the mass resolution, new-type TOF technique has been proposed, using a specific electric field, ‘Linear Electric Field (LEF),’^32,33)^ and actually utilized by several instruments for the planetary plasma observation. The LEF ***E*** varies proportionally to incident ion flight length *z*, as described by ***E***=−*C****z***, where *C* is a constant. In the LEF, the motion of incident ions is expressed by a simple harmonic oscillator and thus the flight time is independent of the mass (see Fig. 3). Although the previous straight TOF technique is capable of measuring ions in the solar wind and Earth’s magnetosphere, the mass resolution is insufficient for discriminating heavy and various ions originating from other planets and moons. The flight time is focused by LEF to a constant that only depends only mass/charge because of the LEF.^32)^ Consequently, LEF TOF mass spectrometers offer considerably high mass resolution. M/ΔM∼20 was achieved by a small-size instrument on the KAGUYA spacecraft,^4)^ and larger-size ones on the CASSINI^34)^ and BEPI-COLOMBO^35)^ spacecraft, which demonstrated M/ΔM >50.

## HIGH-RESOLUTION MASS SPECTROMETERS FOR LANDERS AND ROVERS

For the space plasma observation, ion instruments measures ions in the wide energy range up to ∼10 keV because surrounding electric and magnetic fields accelerate ions. This inevitably causes the limit of the mass resolution. On the other hand, in the case that mass spectrometers on rovers and landers measure neutral gasses originating from the planetary atmosphere and surface, the initial energies are very small around their thermal energies. Therefore, mass spectrometry techniques similar to that used in laboratories are available.

In recent years, the techniques of the planetary landers and rovers have considerably advanced, and thus mass spectrometers were also applied for measuring isotope ratios. The ROSETTA mission employed a double-focusing magnetic mass spectrometer and a reflectron TOF mass spectrometer,^7)^ which detected main volatiles consisting of H_2_O, CO_2_, and CO around a commet.^36)^ The double-focusing and reflectron-TOF instruments achieved mass resolutions of M/ΔM∼3,000 and 500 and sensitivities of 10^−3^ and 10^−2^ (counts/s)/(part/cc) with masses of 16.2 and 14.7 kg and powers of 19 and 24 W, respectively. The predecessor of the double-focusing mass spectrometers is that mounted on the GIOTTO^37)^ spacecraft which also explored to a comet. In addition, the GIOTTO and VAGA missions employed reflectron-TOF mass spectrometers^38)^ and succeeded in measuring light elements.^39)^

In the sample analyses by the Curiosity rover, a quadrupole mass spectrometer with gas chromatograph and a tunable laser spectrometer^6)^ has obtained scientific results such as the isotope ratio of N, C, H, O and Ar.^40)^ Quadrupole mass spectrometers were initially developed for orbiters, early PIONEER VENUS,^41)^ GALILEO,^42)^ and NOZOMI,^43)^ and recently CASSINI.^44)^ The Lunar Atmosphere and Dust Environment Explorer (LADEE)^45)^ and Mars Atmosphere and Volatile Evolution missioN (MAVEN)^46)^ missions are equipped with similar instruments, which have successfully measured volatiles around the Moon^47)^ and upper-atmospheric particles of the Mars,^48)^ respectively. These latest spaceborne quadrupole mass spectrometers have achieved considerably high sensitivity of 10^−3^ (counts/s)/(part/cc). Note that since such high-sensitivity instruments measure gasses emitted from spacecraft, its cleanness and venting are important.

## FUTURE MISSION

For coming space plasma missions, the technique of straight TOF mass spectrometers with top-hat electrostatic energy analyzer has already achieved. Since multi-satellite observation is now going mainstream for the high resolution in time and space as the Magnetospheric Multiscale (MMS) mission,^49)^ the miniaturization of the scientific instruments is the next challenge.

The LEF TOF technique is the most advanced and thus is the most suitable for obiter missions to planets and moons. For the Martian Moons eXplorer (MMX) mission, such a mass spectrometer of M/ΔM∼100 is one of the scientific instruments, which will measure a variety of ion species around Phobos. The mass spectrometer will observe secondary ions emitted from the Phobos surface to conduct ‘natural’ SIMS analyses,^50,51)^ in which the solar wind ions will play a role of the primary ion beam. The LEF TOF mass spectrometer on the KAGUYA spacecraft did such observation of secondary ions from the lunar surface due to the solar wind hitting.^52)^ Ions escaping from the Martian ionosphere will also be measured by the MMX mission, in which isotope analyses will be performed if possible. For these observations, the wide energy range is indispensable because the secondary ions and escaping ions are accelerated by the solar wind electromagnetic fields.^50)^

The era of planetary missions by landers and rovers has started, leading to an increase in demand for isotope analyses by mass spectrometers. In Japan, the development of a spaceborne isotope mass spectrometer using the multi-turn TOF technique^50)^ has started for the future missions, one of which is a landing explorer on a Jovian Trojan asteroid, named Oversize Kite-craft for Exploration and AstroNautics in the Outer Solar system (OKEANOS). The mass resolution is designed to be M/ΔM>10,000 because it is required to implement isotope analyses for hydrogen, carbon, nitrogen, and oxygen which have a variety of hydrogen compounds. Moreover, another Japanese lander for the lunar polar region aiming at observing water is under consideration, in which a reflectron-type mass spectrometer of M/ΔM>100 is one of the scientific instruments. Miniaturization is of course more important for lander and rover missions.

## Notes

Mass Spectrom (Tokyo) 2018; 7(2): S0076
